# Brain Games for Dementia: Do They Help?

**DOI:** 10.1093/geroni/igaa057.2803

**Published:** 2020-12-16

**Authors:** Patricia Heyn, Pallavi Sood, Hannes Devos, Ahmed Negm, Sandra Kletzel

**Affiliations:** 1 University of Colorado Anschutz Medical Campus, Aurora, Colorado, United States; 2 University of Florida, Gainesville, Florida, United States; 3 University of Kansas Medical Center, Kansas City, Kansas, United States; 4 University of Alberta, Edmonton, Alberta, Canada; 5 Edward Hines, Jr. VA Hospital, Hines, Illinois, United States

## Abstract

Brain Gaming (BG) Interventions have been shown to improve the cognitive function of older adults with cognitive impairments (CIs). However, rigorous evaluation supporting BG effectiveness is needed. Thus, we used meta-analysis to evaluate the effectiveness of BG. Several search databases (i.e. Pubmed) were used to identify relevant randomized controlled trials (RCTs). Cochrane RoB tool evaluated risk of bias. The main outcome was the composite score of cognitive function. Inverse-variance random effects model was used to compare the pooled standardized mean difference (SMD) across studies. A total of 16 RCTs included 909 participants. The RCTs varied in sample size, gaming platform, training prescription, and cognition. The meta-analysis showed no significant effects of BG on overall cognitive function (pooled SMD = 0.08, 95% CI [-0.24 – 0.41], p = 0.61, I2 = 77%. However, due to high heterogeneity, we cannot confidently refute that BG is an effective cognitive training approach.

